# Acceptance and rejection of “morally challenging” behaviour in online sperm donation communities: narrative interviews with recipients and donors

**DOI:** 10.3389/frph.2024.1222601

**Published:** 2024-05-21

**Authors:** Georgina C. Forshall, Georgina L. Jones, Rhys Turner-Moore

**Affiliations:** School of Humanities and Social Sciences, Leeds Beckett University, Leeds, United Kingdom

**Keywords:** risks, narrative research, online sperm donation, donor, recipient, artificial insemination (by donor), challenges

## Abstract

**Introduction:**

Online sperm donation allows those hoping to conceive a baby (“recipients”) to meet prospective sperm donors online, via “connection” websites or social media. These sites offer some advantages to clinical donation (including lower costs and greater choice over donation arrangements) but previous research has suggested that these sites may also pose risks and challenges to those who use them. Therefore, the aim of this exploratory research was to better understand online sperm donation communities and the experiences of both recipients and donors, particularly with respect to situations that could be “morally challenging” or involve harm.

**Methods:**

Three prolific donors and five recipients were interviewed using an in-depth narrative approach. Carol Gilligan's Listening Guide was employed to analyse the data.

**Results:**

The findings demonstrated that the donors sought to find ways to maintain autonomy in their donating practices and were concerned about the character and parenting abilities of recipients, as well as the potential for recipients to make unwarranted complaints. The recipients were concerned about their safety and finding a donor they could trust, discussing issues relating to donor “dishonesty”, online abuse, and a lack of support from connection sites and related authorities. Both donors and recipients identified “morally challenging” behaviour relating to donor anonymity (donor use of fake online profiles or aliases) and the sexual motivations or (mis)conduct of some donors. The participants each discussed the ways in which they managed perceived risks.

**Discussion:**

The degree to which the participants voiced their acceptance or rejection of challenging behaviour in online sperm donation communities varied across and within participants, highlighting the complexity of the way in which people interact in this environment. Further research is required to understand how this form of sperm donation can be as safe and supportive as possible, while also respecting the importance to donors and recipients of autonomy and choice when making donation arrangements.

## Introduction

The use of donor sperm as a means to conceive a child has increasingly become viewed as a viable way for single women or same-sex couples to start a family or as solution to male infertility for different-sex couples (1). In the UK, the formal channel open to anyone hoping to conceive with donor sperm is via artificial insemination (AI) at a fertility clinic, regulated by the Human Fertilisation and Embryology Authority (HFEA). Although these clinics offer medical protection (via sexual health and genetic testing) and legal certainty regarding parental rights (2), high costs and other issues associated with both National Health Service (NHS) and private treatments limit their accessibility (3). As of 1st April 2005, clinics have also been legally bound to offer only identity-release donation, whereby donor-conceived children are entitled to access limited information about their donor at age 16 years and more comprehensive personal details at age 18 years old (4). Other forms of donation, ranging from fully anonymous donation to co-parenting scenarios are not offered (5).

An informal system of sperm donation has developed parallel to clinical services, which allows individuals to manage the conception process themselves (6), for example through known or private donation (i.e., by attempting insemination themselves with a known donor or using known donor sperm at a clinic) or by connecting with unknown donors via social media or “connection” websites. The focus of the current study is on this latter route and the specific ways in which recipients and donors connect in online environments. The significant number of people using online sperm donation sites have formed online sperm donation “communities” (7) or social networks (hereafter referred to as communities), overseen largely by the website or social media page owners (ibid). These sites represent a burgeoning industry and are available in many different countries (for example, co-parentmatch.com covers the US, UK, Australia, Canada, New Zealand, South Africa, and Europe), with revenue made from membership fees (e.g., £9.99 per week or £14.99 per month at co-parentmatch.com) or the purchasing of message credits which allow donors and recipients to contact one another (e.g., £30 for 20 message credits at prideangel.com). The amount of people using sperm donation websites worldwide is hard to estimate, however Taylor et al. (3) have suggested that the number of potential recipients based on 60 English language websites and social media pages could be up to 350,000.

Online sperm donation (OSD) offers a broader spectrum of long-term donation arrangements and the opportunity to save money through home insemination rather than undertaking costly fertility procedures (5). Pennings also suggests that OSD de-medicalizes sperm donation and “increases the reproductive autonomy of women in countries in which access to donor spermatozoa is restricted, where rules are imposed that curtail the freedom to build one's family according to one's own values, or both” (8). Despite these advantages, some risks have been associated with the use of online sites to source or supply sperm. These include health risks, such as the possibility of contracting sexually transmitted infections (STIs) or passing on hereditary diseases, and legal uncertainties around parental rights and responsibilities (9). There have also been some safety concerns relating to “morally challenging” behaviour and the potential for abuse of recipients by donors (7). It should be noted, however, that conceiving a child with an intimate partner may carry the same health and safety risks, and that, while using a sperm bank may guarantee the absence of STIs, there is less reliability in terms of genetic testing (5).

While there is significant research on both clinical sperm donation and private sperm donation generally, there are relatively few studies that focus on the OSD context specifically. A principal concern of research on online sperm donors is the characteristics of men who donate via this route and their motivations for doing so (10–16). One of the key findings of these studies is that donors tend to donate for altruistic reasons, which echoes earlier work on sperm donors in clinical settings (17–20). It has also been reported that having greater choice and control over the donation process is an important factor in why men choose to donate via connection websites, along with the potential to engage with recipient families (11), and the ability to receive updates on the lives of donor children (12). Some studies have also highlighted the desire of donors to procreate (10) and the importance of the “symbolic projection of their own procreative identity” (14).

Research on recipients of OSD has focused on why they may seek to find a donor online and the kinds of donors they are looking for. Studies undertaken by Whyte and Torgler (21) looked at the kinds of donors who are likely to be selected by recipients in OSD settings. Their research found that personal characteristics matter more for donor success than physical traits (22) and that recipients tend to prioritise “good character in donor selection” (21). If personal compatibility is at the heart of selection decisions for recipients, this may explain why increasing numbers of women are turning to OSD settings to find a donor (8) as clinics do not provide the opportunity for recipients and donors to meet in person. Indeed, a survey undertaken by Jadva, and colleagues found that 58% of OSD recipients felt that there were advantages to obtaining sperm online, of which 24% further specified that being able to meet a donor was a benefit (6). This is supported in research with lesbian couples considering both formal and informal conception options, which found that recipient parents preferred known donation as it allowed them to “get a feel” for who the donor was (23). Jadva and colleagues (6) suggest, furthermore, that the main benefit for women using OSD is that unlike in clinical settings, they are able to establish relationships with donors on a continuum of contact arrangements, ranging from fully anonymous donation to co-parenting.

Existing research on OSD has, therefore, highlighted the desirability for both donors and recipients of being able to make decisions about sperm donation on their own terms, outside the restrictions of clinical settings. However, one limitation of these studies is that there is little engagement with the social landscape that may lead individuals to consider online sperm donation. The study undertaken by Freeman et al. (11), for example, demonstrates that greater control over who to donate to and how is a primary reason for donors participating in OSD but it does not explore what the implications of this might be for the people involved. In addition, Taylor and colleagues (3) suggest that, in the UK, a lack of access to clinical services is forcing women hoping to conceive to turn to OSD, but the current literature see (6) on recipient experiences does not consider that recipients may first have exhausted all other options before trying OSD. Whilst online sperm donation provides donors and recipients with more opportunities than clinical donation alone [for example, by being significantly more cost-effective or by permitting access for individuals who would have been excluded by clinical criteria; (12)], it is a context that is also defined by a lack of formal regulation and an increasing demand for sperm as a resource in short supply (19, 24). In this environment, it is possible that those who have access to and manage the supply of sperm will have disproportionately more power than those who seek sperm.

The potential for power imbalances in online sperm donation has been picked up on by research undertaken by McQuoid (7), who suggests that the power that donors have in controlling the supply of sperm is putting recipients at risk of abuse. McQuoid undertook three years of covert research by immersing herself in OSD communities, engaging with donors and recipients online and even meeting donors in person under the guise of seeking sperm. Her report indicates that recipients may be at risk of sexual harassment and abuse by online sperm donors, finding that one in two women had experienced physical or sexual assault, trolling, or other forms of harm. She suggests that this abuse is hidden behind stigma, denial, and the complexity of the relationship between donor and recipient. She also claims that the connection websites that introduce donors and recipients do little to prevent these abuses and fail to safeguard their clients. There are several ethical and methodological issues with McQuoid's non-peer reviewed study; however, it does point to the need for further exploratory academic research into online sperm donation communities and any issues that might be putting those who participate in OSD at risk.

Some of these issues are mentioned briefly by other researchers when discussing OSD [see for example (5, 8)]. In particular, Jadva and colleagues (6) found that one third of recipients in their OSD study reported disadvantages to this conception route and, of this number, 40% referred to the existence of “dishonest” donors. A further 11% reported a negative experience after contact with their donor. There is, however, little discussion of what “dishonest” or “negative” might mean, what impact these experiences may have had on the recipients, or how they may have weighed these experiences against the opportunity to conceive a child. In addition, findings from Freeman and colleagues (11) suggest that heterosexual donors using online sites were less likely (than their gay counterparts) to discuss their donation plans with their partners and, of these donors, almost 50% reported “natural insemination” (sexual intercourse) as their preferred method of donation. They also found that “a sizeable minority pursued online donation to facilitate their anonymity and minimal contact with recipient families” (meaning that they did not want to be contactable in the long-term). The authors suggest that this raises regulatory issues but, as with the paper by Jadva et al. (6), it is not clear how these findings may impact the experiences of recipients and their families in real terms.

Our research aimed to better understand OSD communities and the experiences of their members, particularly with respect to situations that could be “morally challenging” or involve harm. It is not the intention of this paper to make a case for or against the existence or usage of online sperm donation; rather it is hoped that, through engaging with the experiences of members of this community, it will open further discussion on the ways in which this form of sperm donation can be made as safe and supportive as possible. We used narrative interview methods to provide opportunities not only to hear the concerns and priorities of individuals in OSD but also to invite an in-depth sharing of their histories, their relationships, and the way they interact with others, as well as the way in which they respond to difficulties they encounter within this context (25, 26). The *Real-Life Moral Choice and Conflict Interview* (27) alongside Carol Gilligan's *Listening Guide Method of Psychological Inquiry* (28) was used to facilitate understanding of how individuals respond to moral conflict in OSD and the way in which they make decisions in those challenging circumstances. This approach (described further in the Method section) was employed in this research to:

1. Provide insights into donor and recipient perceptions and experiences of morally challenging behaviour in online sperm donation communities and the extent to which they feel these behaviours may be normalised within these communities.
2. Explore the kinds of behaviours that recipients and donors accept and that they resist or reject.

## Method

Ethical approval for this study was obtained from the authors' institution.

### Participants

Eight participants (three donors and five recipients) were recruited via social media and through advertisements circulated by connection websites and the Donor Conception Network, as well as a website (A.I. Confidential) that was created for the purposes of the project.

The three donors were invited to take part as they all reported being experienced or prolific sperm donors, had donated to multiple recipients and, as such, had sufficient knowledge and experience of OSD communities to provide in-depth insights into their norms and values. The donors lived in the United States, Australia, and the United Kingdom, respectively. Each of the donors described themselves as being white, heterosexual, and as having no religion, and they were aged between 28 and 36 years old. All had participated in further education and were currently employed, describing themselves as comfortably or reasonably well off. Two of the donors were single and one was in a relationship. They each had tens of donor-conceived children and two reported having their own children (for whom they had parental responsibility). The donors were given the pseudonyms JC, Sam, and Ed.

Recipients were invited to participate if they had had a “less than positive” experience of online sperm donation, and recruitment was open to anyone who had obtained sperm (or had attempted to) from an online donor. The recipients were from several countries, including Germany, Canada, Poland and the UK, but all had had some contact with online sperm donation in the UK. All the recipients described themselves as being white and all but one, who was Jewish, had no religion. They were aged between 38 and 47, had participated in higher education, were in employment and were comfortably well off. Four were heterosexual and single, while one was in a same-gender relationship. Two stated that they had one child each, while the rest had no children. The recipients were given the pseudonyms, Kate, May, Charlotte, Sarah, and Ann.

### Data collection

Semi-structured interviews were undertaken, primarily drawing on the “Real Life Moral Choice and Conflict Interview” (27). The “Conflict Interview” was devised by close colleagues of Carol Gilligan and the interview schedule is designed to work in conjunction with Gilligan's “Listening Guide” (28, 29, 30)—discussed further in the following section on data analysis. The questions in the “Conflict Interview” ask the respondent to consider a situation in which they have faced a moral dilemma and to consider any conflict they might have felt in that scenario (27).

In the present study, rather than in *any* situation, we were interested in specific situations within OSD where the participants may have faced a moral dilemma. We therefore modified the Conflict Interview so that at the start of the interview the participants were given the opportunity to tell their OSD stories in their own words and in as much detail as they wanted to share; this was an open question, designed to gather a holistic view of their experiences before asking specifically about any that might be morally challenging. They were then asked to describe an observation or interaction within OSD that they felt was challenging or resulted in them having to make a difficult decision. The interview schedule for the donors and recipients varied slightly in that the donors were asked about moral dilemmas that they had observed, based on their extensive experience within the community, while the recipients were asked about what they had directly encountered or experienced.

All participants were then taken through the remaining questions of Brown et al.'s (27) original Conflict Interview, including: “*Was there any conflict for you in that situation, such as some form of a dilemma or emotional uncertainty?*” *and* “*Why was it a conflict?*”. The Conflict Interview also allows participants to consider the impact that the situation had on them personally and on others, and to discuss the extent to which they felt the situation represented a moral issue. For example, they were asked “*What was at stake for you in this dilemma? What was at stake for others? In general, what was at stake?*” and “*Do you consider the situation you described as a moral problem?*”

If time allowed, we repeated these steps to give the participants the opportunity to discuss a second moral dilemma they had experienced in an OSD context. Finally, the interviews were concluded by giving the participants the opportunity to reflect further on anything that had been said and by reviewing a positive take-home from the discussion, i.e., the benefits of participating in the research or any happy outcomes from the participant's sperm donation journey. Appendices 1 and 2 provide the modified donor and recipient “Conflict Interviews” in full.

The “Conflict Interview” was used principally as an interview guide and there was some variation in the questions asked for each participant to allow flexibility in inquiring about individual experiences and to enable participants to talk about the events or issues that mattered to them most. Wengraf's Biographical Narrative Interpretive Method (BNIM) (31) was incorporated to probe the participants' answers to the interview questions. This involved making a note of key words or phrases that the participant used verbatim and using these as the basis to frame subsequent probing questions, allowing the researcher to stay true to the intended trajectory of the respondents' narratives.

The interviews were conducted by the first author and took place via online video conferencing software during the period of July–September 2019. All participants were provided with a *Participant* Information *Sheet* and completed a *Consent Form* prior to the interview, and they were given a verbal debriefing and a *Debrief Sheet* at the end of the interview; links to support organisations were provided in both the Information and Debrief Sheets. Each interview lasted around an hour to an hour and a half, was audio-recorded and transcribed verbatim.

### Data analysis

Analysis of how participants identified and responded to moral dilemmas in OSD drew on the work of Carol Gilligan and colleagues [see, for example: (28, 30, 32–38)]. Gilligan first laid out some of the key principles that came to embody the Listening Guide in her book, *In a Different Voice* (30), in which she made the case for an unprecedented model of moral development centring around the notion of both “justice” and “care” as two distinct moral voices. Gilligan urges researchers not to confuse voice with theme, arguing that the voice is not metaphorical but rather is embodied, an instrument of the psyche, and embedded in social and cultural worlds (32). The particular benefit of listening for voice rather than constructing themes is that it allows researchers to zero in on the counterplay of different voices in a person's narrative—such as the interplay of justice and care voices in a person's interpretation of moral conflict and the choices they make (ibid). As such “the voice” is not a singular concept; rather, the self can encompass several voices which are at times complementary and at others in conflict. The Listening Guide is therefore designed to provide a “qualitative, relational, and voice-centred” narrative approach that enables researchers to observe the complexities of individuals’ voices, to hear the significance of both what is voiced and what is silent and to “unearth” trends or insights more comprehensively than other means of analysis (38).

The Listening Guide may be adapted for the purposes of any narrative study, but it provides distinct benefits for researching moral dilemmas (28), and so in this study, it was applied to find out about donor and recipient perceptions and experiences of morally challenging behaviour in online sperm donation communities. In line with the Listening Guide, the first author performed four “listenings” of the conversation. Each listening focused on a different dimension of the narrative: the context of any moral conflict; the narrator's sense of self; and their articulation of two contrapuntal or conflicting voices, which in this study, were the voices of “acceptance” or “rejection” within the participants' stories. This analysis was then discussed and finalised with the other authors. Owing to the limited length of this article, the findings for the final two listenings will be focused upon here, with reference to narrative events provided by the participants.

## Results

When asked about interactions or behaviours that they had observed or experienced in OSD that they felt were challenging or resulted in them having to make a difficult decision (“morally challenging behaviours”), the participants' stories centred around three key issues: (1) concerns around being able to trust other people involved in online sperm donation; (2) sexual motivations of donors within the community; (3) managing anticipated risks. Analysis of the way in which participants voiced their stories revealed varying degrees of conflict in the way they perceived these issues and provide insights into the extent to which they felt able to accept or reject the behaviours or situations they encountered.

Trust was a key concern for both recipient and donor participants, particularly with respect to the way in which members of the OSD community represented themselves online. Both groups of participants talked about the way in which some OSD donors attempted to maintain their anonymity through the use of aliases and fake profiles. For the recipients this represented a challenge as they could not be sure who they were dealing with, and they felt conflicted about the extent to which they could trust these donors. The donor participants felt that anonymity was both impractical and represented a moral issue, particularly with respect to the rights of donor children. Trust also played a role in how the participants identified potential matches with whom to conceive. The donors were predominantly concerned with finding suitable recipients with whom they could entrust their genetic material, while the recipients identified certain types of donor dishonesty that they hoped to avoid.

Both the recipients and donors referred to the sexual motivations of some donors as being a contentious issue within the OSD community. Whilst many of the participants perceived sexual motivations to be inevitable (i.e., that men are biologically hardwired to seek sex), there was significant variation in the extent to which this was voiced as being problematic across the participants' testimonies and there was also a degree of conflict concerning this within some of the individual narratives. For example, there was some disagreement amongst the donors regarding whether sexual motivations were a moral issue for the community or merely a private matter between individuals. The recipients, meanwhile, identified that donors seeking sex was challenging and it was evident that many felt conflicted about whether to accept or resist the sexual behaviours that they encountered, although the extent of this varied amongst participants.

When asked about morally challenging behaviour in online sperm donation settings, the participants discussed a number of risks they associated with OSD communities. For recipients, these related to potential risks to their health and safety and to OSD website or social media use. For the donors, the risks they perceived related to recipients making complaints about donors or recipients ceasing contact with them (“ghosting”). Both the recipients and donors discussed their attempts to mitigate risks, highlighting conflict around the acceptance or rejection of risk, and also their views on the (in)capacity of external regulation to ensure safety within OSD sites.

The following sections elaborate on each of these three key issues.

### Trust: *“Do I really want to get involved with somebody I cannot trust in the first place?”*

The participants talked at length about the people that they had met in OSD communities and their experiences of trying to find an appropriate donor or recipient with whom to conceive. Both the donors and recipients discussed their experiences of trying to find someone they could trust and their concerns around getting a proper sense of the person that they were engaging with.

One difficulty that both groups of participants highlighted was that of donor anonymity, which was maintained through the use of aliases or fake profiles on connection sites and social media. This was especially challenging for the recipients who reported that being unable to be sure of who donors were, and the potential that donors may use aliases as a cover for problematic behaviour, posed a security risk for them both online and in other types of interaction. Sarah said that, on Facebook in particular, she had little faith in the identities presented by donors, explaining that often the donor's profiles looked like they had been set up purely for the purpose of donating and did not depict who they “really” were, “a bit like catfishing”:

*You didn't know who you were talking to, it's like people had set up, if you go to click on a donor, there wasn't any pictures of them or if there was a picture of them, it’d be like pixels. You couldn't even see them properly. And it wouldn't match the profile. So, it puts you off really that they're bogus profiles, just to do like their donations and things.*

Whist getting to know prospective donors was very important to the recipients, they had many concerns about the implications of engaging with donors who misrepresented their identities, and they perceived this as a moral issue because they felt that it could enable donors to act without regard for consequences. For example, they worried this might lead the donor to be dishonest about issues such as their sexual health and family histories, or whether they might use their fake identities to harm them in some way (i.e., by sending abusive messages online). Many of the recipients accepted that donors may wish to remain anonymous to any subsequent donor children and cease communication after donation, but they still wanted to get to know the donors to ensure they were conceiving with someone they could trust and so they rejected prospective donors who they felt were using fake profiles.

The donors in this study also referred to “donor anonymity” as an issue for the community, identifying both the moral implications of this and the unsustainability of this practice as new forms of technology emerge. The donors themselves said that, for the most part, they were happy for donor children to know who they were and to keep in contact with recipient families. They did not want to play a parental role in the children's lives but enjoyed receiving updates and were happy to be contacted by recipients, for example in medical emergencies. They explained that this set them apart from the norm within the community, which was for most donors to donate anonymously. JC attributed the numbers of donors using fake profiles on social media or connection websites to the possibility that “a lot of guys are married” and may be reluctant to tell their significant others about it (“So, they don't want to, you know, start a drama or anything”) or that they may be worried about being identified by someone they have a personal or professional relationship with.

Ed was critical of the reliance on anonymity in both online settings and within clinics. He said that this had neither ethical justification nor made practical sense. He was concerned for the rights of donor-conceived children to be able to trace their biological identity and he referred to the UN Special Convention on the Rights of the Child, which he said stipulated that “we have a right to identity and part of that is knowing who your biological parents are.” He also explained that with the emergence of genetic profiling websites such as 23andme.com, it is now possible for donor children to connect with their genetic relatives, regardless of whatever arrangements had been put in place at their conception. As such, he argued that attempts to maintain anonymity would become increasingly futile as these technologies progress.

Sam felt that it was important for his recipients to know who he was, but he also hoped for the same level of openness from the people that he donated to, particularly so that he could keep accurate records but also because receiving updates on donor children's progress made him feel as though the “gift” he had given was worthwhile. His approach to donor anonymity could be characterised as “live and let live”, in that he didn't think it was for other people to pass judgement on donors” approaches to donating, as long as they were being honest about their donating intentions (i.e., methods of insemination and so on). However, his attitude towards recipients was more stringent. He felt that donors had a responsibility to find out as much about the recipients as needed to assess their appropriateness to be a parent and to safeguard the wellbeing of any future children. He advised:

*You don't want to have a child coming back to you at 18 and say, I had a miserable shit upbringing, you know, because the point of helping people in creating life is for a positive note. Why would you? You know, it defeats the purpose, hearing about someone being brought up in traumatic circumstances.*

This highlights the second difficulty that both groups raised: there were not only concerns about being able to trust who the person was, but also about being able to gather a clear sense of the kind of person that they were or of their personal and life circumstances. The donors all advised that they had put screening processes in place to decide whether they would be prepared to donate to the recipients who got in touch with them. This contrasts with the clinical route where recipients select donors on the basis of particular characteristics and the market availability of the sperm they would like to select/buy. Before agreeing to donate to recipients, the donors asked them questions online or in person or gave them an “information pack” and asked them to complete a questionnaire. The donors' main concerns related to recipients' health and lifestyle choices, as well as their financial security.

Both Ed and Sam advised that they used social media to screen recipients by looking up their profiles and making assessments about their lifestyles. Sam provided some insights into the approach he took:

*Oh, of course. Yeah. Like some people that are constantly up at 3:00 AM in the morning and sending messages, which aren't working night shifts and that, so you’re thinking, okay, what's their lifestyle like being up that sort of time and yeah, like there's pictures of them drinking copious amounts of alcohol or with massive pupils and stuff like that. So yeah, you do get a bit of an insight, you know, if someone walked into a clinic, they’re not obviously going to go in drunk and that, so that the clinics are going to be able to see that. Whereas on social media, you get a better in-depth view of sort of their life and their lifestyle.*

In addition to smoking and drinking, the donors referred to weight as a key health and lifestyle issue. JC advised that he preferred not to donate to women who he deemed overweight because “usually bigger women are more likely to have health complications during pregnancy.” He did not turn these women down directly but often suggested that they tried to become “healthier” and lose weight before they started the donation process. Similarly, Ed explained that what was important to him was not the quantity of recipients, but rather “it's very much about the quality of the recipient.” He advised that: “I once got into a lot of trouble on a group saying that I want to donate to people who are a healthy weight.” He recognised that this is a very emotive subject and he said that he no longer stated publicly that he screened his recipients for this, but that he took this into consideration privately.

As well as health and lifestyle screening, the donors assessed potential recipients' financial situations, advising that they would not donate to someone who they felt could not afford to have children. As JC explained:

*Well, in my perspective, there's a lot of people that can’t afford a kid. I mean, I’ve had women hit me up that are on welfare […] Some women don’t have the ability to afford a kid […] And there’s a lot of other women that are living month to month. They can’t have kids. Um, I typically try to screen the women to make sure that they can afford the kid. […] That’s a big concern, I think. In the community.*

The donors also wanted to avoid a situation where they themselves might be held financially liable for any donor children at a later date. JC said that he did not think the government would take a sympathetic view to a contractual arrangement between a donor and a recipient where the donor had helped a recipient conceive who was seeking welfare support from the state. In such circumstances, he was concerned that a donor would be required to pay child support.

JC acknowledged that screening recipients sometimes caused issues in the community, particularly as some recipients did not feel that the donors should have the right to determine their suitability to raise a child and, in some cases, they complained about how they had been assessed. However, he felt that recipients did not have a right to challenge a donor's decision to refuse to donate on these grounds, arguing that women who complained did not understand the values of the community and that they were being “selfish”. Ultimately, he was of the opinion that donors should have “an absolute say” regarding how their sperm would be used, but if they agreed to donate to unsuitable women, they should take responsibility for any adverse outcomes. He compared the donor's position to that of the recipients, stating that recipients are not required to use a particular donor if they do not want to, and so donors should also have the right to choose who they donate to. He summarised: “No one can force someone to do what they don’t want.”

The recipients, by contrast, spoke less about the specific criteria they may have had for choosing a donor, and were instead more concerned about avoiding potentially “dishonest” donors. This was a fundamental moral issue for the recipients, and they discussed, from personal experience, a number of ways in which they had encountered donors misrepresenting themselves. For example, several recipients referred to experiences with donors who had not been truthful with their own families, and particularly their wives, about their donation practices. One donor told Kate that he was interested in becoming a co-parent, but she gradually discovered from their conversations that he was married, and his wife (who had recently had twins) knew nothing of his intentions to donate or become a co-parent. She explained that it was “baffling” to her to try to understand or empathise with what drove donors to act in this way. Charlotte meanwhile found one man who she thought might make a suitable donor. He lived nearby, was married with children, and had become interested in donating after seeing a documentary on television. After they met, his wife became uncomfortable with the idea, however, but the donor “felt guilty”:

*…then he tried to keep in contact, and he was like, you know, it's your fertile period, I can come around anyway. And I just said, look, if your wife's not happy, don’t be doing this and put a strain on your relationship and have a secret, just don't do it.*

May also got in touch with a donor who informed her that he had not told his wife about donating and this did not sit well with her:

*But what turned me off was, I mean, he was honest with me about this. So that was good. But he was a bit older than me, and he had a long-term partner, and she was a bit older as well, so she’d had maybe a couple of IVF rounds, and it hadn’t worked. And she didn’t want to do donor egg, so they had kind of called time on their fertility journey I guess, and so he was open with me about all of that, but, and he was also open with me about the fact that she didn’t know that he was doing all this, like, meeting.*

Following these experiences, the recipients each deselected the donors they had met on the basis of not wanting to “start out on a lie” (Kate), to “put a strain on [someone else's] relationship and have a secret” (Charlotte), or because they felt concerned for any third parties involved in that situation (May). The recipients explained that these were not the kind of relationships that they wanted. Kate, especially, was frustrated that in her search to find a co-parent and someone that she could share parental responsibility with, she instead came across many donors who wanted to make a commitment only as far as the donation itself, and often without the knowledge of their own families. In addition, the donor with whom she conceived her child agreed to what she thought was an exclusive co-parenting relationship, but she later found out that he had been donating to multiple other recipients. She said that his dishonesty about his donating intentions felt like a “huge betrayal”, and she was concerned about the impact on her child of not having the father figure she had hoped they would have and about how they would manage their relationships with the other recipient families.

### Sexual Motivations of Donors: *“He thought he was going to get sex…”*

All the recipients advised that they had a sense that many donors wanted something in return for a donation (“some pleasure or another favour”—Ann) and that often this was sexual. They each explained that they had experienced some form of donor behaviour online or in person that was sexually motivated and that they characterised as being morally challenging. May, for example, advised that she had been sent abusive messages from donors online when she insisted that she was only interested in conception via artificial insemination. Similarly, Ann felt that many donors were only interested in “natural insemination” (NI) and seemed to have a hidden agenda, “ghosting” (disappearing) when AI was brought up. Several of the recipients described instances where donors had tried to persuade them to engage in sexual methods of insemination, by claiming that these methods were more effective. Ann said:

*There was a guy that […] there were several of them actually […] he just tried to describe how it was beautifully naturally conceived for the woman that he met. And that they, you know, and they all worked out very well from the first time. And then he had a couple of other successful experiences, from natural conception and when I looked at him, yes, he was a young and intelligent guy, but there was something there telling me that I don’t want him to be my donor.*

In addition, Kate and Charlotte talked about difficult experiences with their donors in which the donor's behaviour could be defined as sexually coercive or abusive, although they didn't label these experiences in those specific terms themselves. For Kate, this involved feeling like her donor incrementally crossed personal boundaries, such as by asking to stay the night at her house or using her belongings, which she felt was inappropriate given the nature of their relationship. He then suggested that they try having sex to conceive, which she said she wouldn't have considered “if I’d had my clear head on”. Charlotte described how she felt “frozen” when she got the sense that her donor had come to her house anticipating sex despite agreeing to AI, and had then masturbated in front of her, expecting her involvement. Her testimony highlighted the significant degree of conflict she felt about his actions, and she discussed how easily the line between a donor/recipient relationship and an intimate-partner relationship could become blurred.

Kate felt that a lot of the donors on “these Facebook groups” were simply “a bunch of dudes looking for sex” and she rejected the notion that donors were altruistically motivated. She referred, for example, to the “biological urge” that might motivate some donors as something that may be fetishistic, a sexual “turn-on” or an embodiment of an egotistical desire to reproduce their genes:

*…guys want to come, and they want to hit the jackpot and get someone pregnant, and that's the natural urge. Um, I think they do like to think about multiple women, multiple children they're fathering. And a lot of it comes down to ego that they think in some way, you know, they have these special genes, they are hyper-intelligent or they're especially good looking or they're super fertile or you know, they're not gonna leave any legacy, “cause they might have a boring job. Um, nobody appreciates them. So at least they can create a baby or lots of babies.”*

Concerns around the sexual motivations of donors meant that the recipients found it hard to navigate OSD because it was difficult to find donors who seemed to genuinely want to donate or begin a co-parenting relationship. All of the recipients said that when they started looking for a donor online, they were only interested in conceiving via AI. Three of the recipients rejected sexual advances either because they were in a relationship or because they ultimately decided to disengage with OSD and return to thinking about clinical options instead. Kate and Charlotte were, however, more conflicted about the acceptability of their donors' behaviour, partly because they had known their donors for several months and had attempted conception multiple times and partly because of an acceptance that these types of behaviours might be “par for the course” within OSD settings.

Throughout the interviews, the donors discussed their own motivations for donating as well as what they perceived to be the motivations of other donors. Generally, they talked at length about the altruistic motivations of donors and the sense of giving back to the community, but when considering morally difficult behaviour, they referred to the sexual, financial, and ego-driven (as Ed put it, the desire “to have more of me around”) reasons for donating. The donors agreed that sexual motivations were the most contentious within the community but disagreed, however, on the extent to which they felt this was a moral concern or, alternatively, a private matter between individuals.

Ed explained that a community norm was for donors to request “natural insemination” (NI) from recipients: “they’ve [the donors] realized that they’ve got something that women want. And they can use it to get what they want,”. On the one hand, Ed felt that if two consenting adults agreed to conceive by having sex with one another, he did not personally see a problem with that, but he was sceptical of the extent to which recipients were open to such an arrangement. He thought that the recipients were only really interested in obtaining sperm, rather than starting a sexual relationship with the donors.

Ed felt that donors seeking sex from recipients were “combining two things that don’t need to be combined really at all.” He did not personally consider sperm donation to be a means to obtain sexual gratification: “Because for me, it's not a sexual thing at all. Nine times out of 10, it's a lesbian couple in bed alone. Yeah. It's just a coincidence that really for me to get it out, I have to, you know, I'm putting my mind in a sexual kind of place. It's not turned on.” He was unable to empathise with donors who were motivated by the potential of sexual contact with recipients, saying that he could not imagine anything worse than having sex with someone who didn't want to but was complying with the donor's demands as a condition for them to conceive a child. He recalled speaking to one couple who told him about meeting a donor who had agreed to donate via AI:

*And then, at the last minute, I don’t know, he somehow tricked them into it. And they were so, you know, they knew they weren’t going to get anyone else that cycle, they just wanted it to work. She went ahead and she slept with them. So, this is a married lesbian woman, but she did have sex with this guy. It didn’t work thankfully… And I was just kinda like, well, I'm so glad. Because, of course, if your biological father is a sexual predator, if he happily enjoys having sex with someone who he's coerced into having sex then maybe the son would as well, you know, things do get passed down. Um, so why would anyone want a sexual predator as the biological father of their child?*

Sam and JC, however, took a more pragmatic view of the culture of the OSD groups that they participated in. Sam made the point that “everyone's adults and people are making adult decisions,” explaining that he felt that methods of insemination are something that should be left up to individuals to determine, comparing this to the way people conventionally meet and decide to have a child; that it is not something that should be regulated nor the concern of anyone else. JC, meanwhile, explained that “a norm in the community is typically guys prefer sex”. He advised that most donors in the community would only consent to helping a recipient conceive a baby if it was done though NI: “So, there's about 20 per cent of donors that are open to artificial insemination. But honestly, realistically, I'd say easily, at least 70 to 80 per cent of donors will only get you pregnant if you're going to have sex with the guy.” Reflecting on his own experiences, JC said that although he would not try to push sex, it is the method he prefers: “obviously if the woman is attractive, I think in any situation if the guy's attracted to the girl and they’re open to it, of course, I'm going to do that over artificial insemination.” He went on to say that if the recipient is explicit in wanting to conceive via AI, he would help them conceive using this method. However, if they seemed open to NI, he would advise them to try this method, and “most of the time they’re, they’re ok doing that”:

*Natural insemination is more effective. Personally, I don’t want to get into [an] argument with people, but I think it's a lot more effective having sex to get pregnant […] but people don’t want to hear the hard truth because they don’t want to have sex with the guy to get pregnant. But um obviously, if I say it on [redacted], you're going to have women going crazy and attacking you because they don’t want to debate that. But most guys agree. […] And I’ve had my experiences, woman [sic] got pregnant much faster through natural insemination than doing it in a cup, artificial insemination. So, it's more like I don’t want to get in a drama, so I don’t try to push it. Um, I do usually prefer it though.*

He said he felt that many of the donors were up front about the fact that they preferred to have sex rather than donate via a sterile cup, and he didn't think that there was anything wrong with that: “These guys can’t be blamed. Guys are guys. If a girl seems that she may be open to having sex with the guy to get pregnant, you can’t blame the guy for it. A guy's a guy, it's a natural instinct.” For the donors, therefore, the issue of sex within OSD was a complex moral dilemma. For the most part, they accepted that sexual motivations were likely to be an almost “natural” occurrence, in much the same way as sex can become part of intimate relationships. They could see, however, that the sexual behaviour of some donors was negatively impacting recipients in their communities, and this was problematic or had the potential to cause “drama”.

### Managing Anticipated Risks: *“Does everything need to be regulated?”*

Both the recipients and donors discussed risks that they associated with the OSD community and, where they thought it was possible to mitigate these risks, the steps they took to do so. Perception and acceptance of risk varied amongst the participants, however. A common concern for recipients was the risks to their health and safety that OSD might pose. The recipients explained that they did not feel that meeting a donor alone was particularly safe and they took actions to mitigate the risks. For example, many of the recipients opted to meet donors at hotels instead of where they lived, told friends and family where they were going or brought someone with them for support, and emphasized their preference for AI only in advance. They also discussed the possibility of contracting an STI from an online donor given that sexual health testing in OSD is not as reliable as via clinical conception.

However, the recipients tended to compare these risks to those that they might encounter in “real life” rather than in clinical settings. For example, with respect to safeguarding their sexual health, the recipients were happy to rely on requesting sexual health tests from donors and, in some cases, were confident enough in the medical treatments available to them if they were to contract an STI. May hypothesized that the risk of STIs was something that those in even long-term relationships could not avoid:

*Well, you know, my friend who's married, her husband could be having an affair, and she ends up with an STD as well. Like, I actually did think, I kind of probably thought those things so that I could rationalize to myself that, like, it was okay because it was kind of the same risk.*

The recipients also compared the risk of meeting donors in person to their experiences of online dating or other relationships that they had had. The fact that many of the recipients had had experiences with online dating meant that they felt equipped to manage OSD in the same way and they screened potential donors by looking at their profiles and engaging in conversation with them online or over the phone before agreeing to meet them. They felt that these risks were akin to the “everyday” risks that women face in maintaining their personal and sexual safety.

Many of the recipients, however, explained that they had started looking for a donor online after considering all other options to conceive a child. They discussed the end of relationships with intimate partners, disappointing experiences of online dating, fertility issues, experiences of unsuccessful IVF and a lack of access to treatment due to costs or their eligibility for treatment. May felt that women would avoid OSD sites if other routes to conception were not “so prohibitively expensive”—“I can guarantee you this is nobody's first choice, right?” At the point that recipients come to online sperm donation, therefore, they may have been trying to conceive for several years and may have exhausted all other options, meaning that they felt they had to accept risks that they otherwise would not. May explained:

*… the first guy I met and the first time I actually agreed that I was going to go to a hotel and do this. Um, that was like a difficult decision because in no other aspect… I guess with like infertility, whether it's because you're a single woman or because you’ve had problems or both, like my case, it kinda like just makes you desperate. Right? And you do things like never in a million years would you do…*

Specifically, the risks that the recipients identified in relation to OSD sites included a lack of confidence in donor identities, abusive messages, and “ghosting” (discussed earlier). They also reported feeling that there was a lack of official recourse offered by the owners or “Admins” of connection websites and social media pages/groups if “things went wrong” or in situations where recipients had had difficult experiences with donors. Sarah, for instance, had very low expectations of the kind of support that she felt she could expect from site owners. She said she did not feel that it was possible to report abuse on Facebook because of a lack of enforcement from “Admins” regarding acceptable behaviour and a lack of clarity on whether anyone monitors the groups. She did feel, however, that the “Block” function provided a powerful tool that was sufficient to protect herself from unwanted communication from donors. Kate described the community as a bit “Wild West” and she explained that her experiences had left her with little faith in the safeguarding power of website owners, the HFEA, fertility clinics, and other agencies.

Whilst the recipients expressed their disappointment about the perceived lack of support, all but one of the recipients accepted this as something to be expected from these specific sites. They either attempted to manage the risks of OSD themselves (Sarah and Charlotte) or they disengaged from this type of sperm donation entirely by closing their online profiles or accounts (May and Ann). Kate, by contrast, attempted to report her concerns about her donor to the OSD sites she used, as well as fertility clinics (because she suspected that he donated to these also) and the HFEA. She said that the people she spoke to were either unwilling or unable to help, in part due to a lack of specific powers to do so and in part because of her donor's ability to simply create a profile under a new name if his existing one was shut down. In the end, she was able to successfully seek recourse through the Child Maintenance Service to ensure that the donor made payments towards the care and upbringing of her child.

Given the risks identified by the recipients and their perception of the current lack of recourse available to them, they felt that additional regulatory safeguards should be put in place to protect people who engage with OSD from harm or abuse. They did not think, however, that the connection websites and social media pages should be “banned and shut down” because they remain one of few options for many women to have a baby. May said that if she were “10 years younger with no fertility problems”, she would probably still be using connection websites despite the negative experiences she had had because of the lack of other conception routes available to her.

The donors’ key concerns around risks related to the possibility of recipients “ghosting” them and of recipients making complaints. By “ghosting”, the donors explained that they had encountered recipients with whom they had had significant contact who had then blocked them or stopped replying to messages or who had successfully conceived and then had, in effect, completely disappeared. The latter was of particular concern to Sam who valued receiving updates about donor children as a reminder of why he had made the “gift” in the first place. It was important to him to know that the children were being well looked after and that his donation had been appreciated. All three donors also kept records of the people they had donated to and so they felt some degree of contact after conception was important for this reason. There was an acceptance amongst the donors, however, that “ghosting” came with the territory of OSD, although they attempted to avoid this through careful screening prior to donation (discussed above).

More troubling to the donors was the potential that recipients might make complaints about them or what they perceived to be the prevalence of recipients making unwarranted complaints about donors in OSD communities. On a personal level, they had encountered recipients who were not happy with the way they had been screened or the amount of expenses they were being asked to cover, but more generally on OSD sites, they had observed recipients complaining about being asked to engage in sexual methods of insemination. JC identified donors “pushing” recipients for sex as one of the key complaints from recipients in the sperm donation community: “I think every donor at some point has been accused of pushing it.” To some degree, Sam and JC accepted the legitimacy of these complaints but felt that they might be unjustified if the recipient had not expressly stated their preferred method of insemination from the outset. Sam, for example, explained that one of the rules of the OSD group he was in was to state preferred conception methods in personal advertisements. JC also advised:

*And, well, you should have stated that you only wanted artificial insemination. You need to be up-front; I always tell people up front. [redacted] you must state the method you want. The reasoning is because a lot of times when they don’t state, oh I want to do artificial insemination only, you have men that contact them that will only do natural insemination. And I always tell them, well this is your fault. You should be stating that you only want artificial insemination up front*.

JC advised, “It's just really women that are complaining, you know”. He did not feel that a recipient had a right to complain about donors asking for sex, if she is “open to the idea of doing natural insemination” (although he did not specify how he determined this). He also did not think that recipients should complain about donors publicly, rather that these issues should be handled by the individual members of the groups themselves. As an online Admin, JC did not allow complaints within the community he oversaw and blocked or muted recipients who complained in a manner that he felt to be unreasonable.

The donors differed in their views about whether, how and the extent to which OSD should be regulated within the sites themselves or by external authorities. On the one hand Sam and JC were strongly against the idea of legal intervention and felt that community members have the ability to “regulate themselves”. On a personal level, Sam said that he felt that the way he managed his donating was “pretty regulated” (in terms of screening and record-keeping) and, of the community more generally, he advised:

*…in terms of regulations, I mean, does everything need to be regulated, you know? Say like when you go out shopping, do you need to be regulated to make sure you're putting stuff in your shopping trolley with the correct posture or, you know what I mean? If people have the information in front of them and the community support around them, you know, it's sort of a warming feeling [when] people get shown the ropes. Uh, you know, that's its own regulation…*

JC agreed with the idea that people should be responsible for managing their own sperm donation activities themselves, arguing that individuals should navigate OSD communities in any way they wished: “If you don’t like someone, you could easily block them. People are stupid. Some women are lazy. They're like, Oh I don’t want to block this person. Okay, well you know, if you don’t like the person talking to you, you just block them.”

By contrast, Ed felt that in order to promote and expand the online sperm donation community, more regulation was required, and he felt that the HFEA should be playing a bigger role in providing guidance to recipients and donors opting for the online route. He was keen that new donors with good intentions “don’t fall into these traps where they can get into a lot of trouble later on” but he felt that the government could be providing more guidance to those wanting to conceive with donor sperm outside of a clinic. He listed some of the possibilities for a more regulated online sperm donation community:

*And, you know, even a registry where a donor would have to get checked every so often or you know, that you could regulate this without making it into a bad thing and just getting people thinking about, you know, even, registering the details of the child. There's no reason why the HFEA couldn’t, if they were funded properly, couldn’t regulate donating and I'd be quite happy to take part in some kind of regulated system.*

In the absence of formal guidance and regulations, Ed thought that the current way in which OSD sites police themselves could sometimes be “unfair” or heavy-handed and that this was problematic, especially as blocking donors and recipients from groups could significantly impair their chances of donating to or conceiving with someone. Ed also felt government regulation was important to bring the notion of sperm donation into the mainstream and to raise awareness of the possibilities of OSD. He rationalized that increasing the number of donors available to recipients would empower them to reject donors they were not comfortable engaging with and, consequently, would limit the scope for abuse.

## Discussion

The aim of our research was to provide insights into donor and recipient experiences of “morally challenging” behaviour in OSD communities. We were particularly interested in finding out about whether participants felt that morally challenging behaviours were normalised within OSD communities, and if they personally accepted or rejected these behaviours.

Perhaps the most striking aspect of the donor and recipient interviews was the diversity of experiences that had led each of them to consider participating in online sperm donation. Despite differences in personal histories, there were, to some extent, commonalities in how the participants described their experiences of being involved in OSD communities and the aspects of these communities that they perceived to be problematic. As qualitative researchers with experience of thematic analysis, it was challenging to shift our focus from thinking about our participants' experiences in terms of common themes and to instead learn to listen for the interplay of different voices that were embedded in their narratives, as Gilligan's guide (28, 29, 30) recommends. However, by doing so, we were able to identify not only the moral issues that were of key concern to the participants, but also by listening for the contrapuntal voices of acceptance and resistance, we were able to unearth some of the complexity around how they perceived and experienced morally challenging situations or behaviour within OSD contexts.

### The recipients

The recipients discussed a number of moral dilemmas relating to who to trust in OSD, the potential for donation to become transactional (that donors wanted something in return), and how to manage risks to their personal safety. The morally challenging behaviour that they identified included donor use of fake online profiles or aliases, donors being dishonest with their families or about their donating intentions, the sexual motivations or conduct of donors, online abuse, and a lack of support or recourse from OSD sites and related authorities. Their ability to accept or reject what they perceived as challenging or difficult behaviour was complicated by their own desire to have a baby, the nature of the OSD communities and the broader context of access to regulated fertility treatments and funding.

These findings lend some support to claims made in previous literature that recipients value the additional choice over conception and contact arrangements that OSD offers (6, 8), as well as personal compatibility and the ability to meet donors in person (21, 22). However, the narratives of the recipients in this study demonstrate that the issue of donor anonymity was a significant concern for them and that at the forefront of their search for a donor was trying to find someone they could trust. Govier (39) argues that trust is an issue often overlooked by social contract theorists and that forming trust in relationships is a complex process. She explains that when we trust someone, we base our decision to do so on our perception of that person's motivations and competence; that is, whether we think that person will act well and not cause us harm and whether they have the necessary knowledge and experience for what they assert (40). As such, the process of obtaining sperm online goes beyond simply an exchange or transactional agreement for the recipients. The trust they need in these situations involves establishing that the donor can do what they say they will do (i.e., to help them to conceive and, perhaps further, to conceive a child with specific attributes or characteristics) and also gaining the confidence that the donors will behave in a way that is not harmful to them or their future children. However, as Govier argues, trust is “fundamentally an attitude based on beliefs and feelings, and implying expectations and dispositions” (41). This poses a problem for those seeking connection with strangers in online spaces due to the need to base their belief of someone's trustworthiness on evidence. If they are uncertain of the evidence (for example, a person's profile picture or given name), it is therefore much harder to trust.

The recipients were particularly worried about the safety implications of meeting strangers from the internet or conceiving with someone whose real identity was unknown to them. Their stories add depth to the mention of “dishonest” donors in the study by Jadva and colleagues (6) and the finding in Freeman and colleagues (11) that there is a tendency amongst heterosexual online donors to not disclose their donating practices with their partners. The recipients talked about their encounters both with donors who concealed their plans to donate from their families and with donors who had not been honest with them about their donation intentions. They talked about the negative impact that this had on them, the harm that they felt this might cause their donors' partners, and the implications this may have for any donor-conceived children. It was important to them to “not start out on a lie” and they rejected donors who they perceived to be dishonest and who they felt they could not trust.

The narratives of the recipients also provide further explanation about what might be meant by “negative experiences”, mentioned in previous research (6, 8). The recipients discussed experiences of online abuse, as well as behaviour that could be interpreted as sexual abuse or coercion in person. This supports the findings in McQuoid's (7) research, that recipients may be at risk of harm such as sexual assault. It is evident from the recipients' stories that they prioritised identifying safety and trustworthiness in their donor over other selection criteria. These are traits that were not considered in Whyte and Torgler's (22) survey study, which drew conclusions on the types of OSD donors that recipients looked for by asking donors about their demographic and personality characteristics and measuring donor selection success with the number of donor-conceived children they had helped to conceive. The study found that intellectual, shy, and systematic donors were more likely to be selected by recipients than lively or extroverted donors. However, without directly asking recipients how they choose donors, it is difficult to determine the extent to which they might find prospective donors to be risky or threatening.

Existing research suggests that there are strategies available to recipients to manage risks or respond to difficult donor behaviour. Gilman and Nordqvist (5), for example, advise that while health risks are higher in OSD, “the use of [STI] testing can arguably reduce them to then a lower level than might be expected in a typical “natural” conception’ (8). Pennings also suggests that a recipient who finds donor behaviour unacceptable “should cancel the deal”, although he acknowledges that, “In reality, things may not be that simple” (8). We can see some evidence of these strategies in the experiences of the recipients in this study. They carried out risk assessment and management activities when engaging with donors, for example by requesting the results of STI tests, being upfront about wanting AI only, and putting in place personal safety measures by meeting donors in public places or bringing along someone for support. The recipients also advised that they disengaged with (or “blocked”) individuals or the community altogether (by closing online accounts) where they perceived the risks to be too great. This is something that the recipients felt to be consistent with an approach they might take (or they saw their friends taking) to “everyday” risks.

Inherent in such an approach to risk, however, is the assumption that recipients should shoulder the responsibility of keeping themselves safe. Vera-Gray and Kelly write that “women and girls globally are routinely making strategic decisions to avoid sexual harassment and other forms of sexual violence” (42) and they discuss how “safety work” is the “invisible work of being a woman” (ibid, p. 268). This represents a broader cultural narrative which expects women to be responsible for preventing the (sexually) aggressive behaviour of others, and which tends to blame women for their own sexual assaults (43). This narrative has come to be known as “rape culture”, whereby sexual violence is normalised, and victims/survivors are blamed for their own assaults (44). Whilst men are more likely than women to demonstrate a higher acceptance of rape culture (43, 45), women have been shown to internalise the message that the onus is on them to prevent sexual assault (44).

Further complicating the extent to which the recipients accepted or were able to reject challenging behaviour in OSD communities is the broader context in which they have had these experiences. First and foremost, all the recipients had a strong desire to be a mother and conceive a child of their own. They had each faced barriers to other forms of donor insemination (due to restraints on eligibility for funding or criteria for treatments) and the four heterosexual recipients had tried to find an intimate partner to conceive with prior to thinking about finding a sperm donor. The recipients were aged between 38 and 47 and they discussed feeling that time was against them in terms of their fertility. Some recipients had also experienced fertility issues such as polycystic ovary syndrome or recurrent miscarriages. As May put it, OSD is “nobody's first choice”. This meant, as the recipients explained, that they felt they had to tolerate some undesirable behaviour if it meant that they stood a chance of being able to conceive. The conflict experienced by both Kate and Charlotte when considering the sexually inappropriate behaviour of their donors is demonstrative of this, given that there was so much at stake for them in these relationships.

Such circumstances can put recipients in a vulnerable position, and this casts some doubt over the ease in which they are able to mitigate risks or opt out of situations that are problematic for them. McQuoid (7) suggests that some online donors may be aware that most recipients lack other viable options for conception and are conscious of the unequal position this puts women in; a position that some donors might then take advantage of (a view that was also verbalised by Ed in his interview). Research on sexual consent, moreover, has demonstrated that power inequalities implicitly constrain individuals' freedom to consent (46). When considering the decisions recipients make in OSD, therefore, it is important to consider their particular position within these communities; it is possible they may have disproportionately less power in determining the kinds of behaviours that they will “accept”, and they may have few alternatives available to them.

The findings of this research have demonstrated that recipients may also be disempowered by a perceived lack of recourse from both OSD site owners/admins and by a lack of regulatory safeguards. Given the access issues to other routes to conception explained above, the recipients did not want to see an embargo on OSD sites, but they did highlight the need for greater protection. Their experiences echoed research undertaken by Nakata and colleagues, whose Japan-based study found that 96.4% of the OSD websites they reviewed were unsafe for recipients (47). They identified that missing, false or ambiguous information about site owners or representatives poses a risk to recipients (ibid). The findings in the current study also demonstrate that a lack of clarity over who to report challenging behaviour to impacts the recipients' ability to take action and, furthermore, that they had little faith in the safeguarding capacity of external authorities, such as the HFEA.

### The donors

When asked about any morally challenging behaviour that they had observed in OSD, the donors' primary concern related to the importance of being able to determine the “quality” of recipients and to decide for themselves who to trust with their donations. They also discussed donor anonymity and sexual motivations, as well as their concerns about recipient “ghosting” and complaints. They identified a number of community “norms” relating to these issues but varied in the extent to which they accepted or rejected these types of behaviours.

The donors' narratives firstly provide support for findings in the existing literature that donors value the ability to get to know prospective recipients and potentially any donor-conceived children (11, 12, 14). This is evident in the screening processes that the three donors had in place when choosing recipients to donate to, and their concern for donating to recipients who could afford the expense of a child and who could provide them with a “good” upbringing. They emphasized their prerogative to screen because they each felt that they had a vested interest both in how their genetic material was going to be used and who was entitled to use it. While none of the donors played a prominent role in the daily lives of their donor-conceived children, it was still important for them to receive updates about their progress, both for record-keeping and, also, as confirmation that the “gift” they had given was worthwhile. Research undertaken by Riggs and Sholtz (48) on private sperm donation (i.e., between donors and recipients who have met outside of a clinic or who know each other already) similarly identified that donors perceived sperm as a “marker of genetic legacy”, which allowed them to “leave their mark on the world” (p. 46), and sperm donation as a “gift” to others (p. 52). They also found that donors were mindful of the rights of donor-conceived children and wanted to donate their genetic material responsibly.

Although the capacity to screen recipients was a high priority for the donors, aspects of their screening processes may be problematic for recipients. Donor screening in OSD vastly differs from sperm banks and fertility clinics, where the recipient's choice of donor depends on the market availability of the sperm. In such scenarios, the only qualifying factor for recipient eligibility relates to whether they can afford the treatment on offer (49). By contrast, in OSD, donors have the opportunity to decide which recipients they deem fit to be mothers. When making these assessments, the donors factored in the health and lifestyle of the recipients based on information that they had gleaned through conversations with the recipients and from their social media pages. Of key concern were the recipients' weight, smoking and drinking habits. Arguably, the assumption that these characteristics or behaviours may impact a recipient's parenting capacity relies upon gendered stereotypes of mothering. Kobrynowicz and Biernat write that social labels attributed to being a “good” mother include “cares for her children before her”, “would keep the house clean” and “instills “family values”, while labels applied to “bad” mothers include “doesn't do everything possible”, “very lazy”, “drinks too much” and “she is selfish in that she places her happiness over the happiness of the child” (50). However, the notion of the “ideal mother” is problematic in that it infers a “standard of motherhood [that] is impractical and unreasonable and punishes those who fail to meet its criteria” (51).

Jackson and Mannix (52) define “mother blaming” as a “pervasive and serious problem” that “complicates the already complex responsibilities that comprise mothering” by “attributing problems with (even grown) children to maternal fault” (p. 150). In the current study, the narrative of “mother blame” (53) was particularly evident when the donors appeared to conflate weight with health, assuming that larger women were less healthy and less likely to participate in healthy activities. Conversations around “maternal obesity,” as well as a general cultural tendency to “fat-shame” women (54), have been identified as resulting in a new form of mother blame that imagines women genetically passing on their “obesity” to their progeny (55). The overweight mother is then culturally perceived as toxic, producing children of “lowered quality, in terms of health, behaviour or achievement” (56). The donors' screening practices may therefore be influenced by cultural narratives of weight that determine the kinds of women who they allow to be mothers.

Furthermore, for the most part, the donors thought autonomy over their donating practices was more important than the prospect of further regulatory intervention in sperm donation, a finding that lends weight to the claim by Freeman and colleagues (11) that donors value the greater choice and control that is offered by OSD. One donor could see a place for regulation to limit “morally challenging” behaviour while the two other donors took a more individualistic or libertarian approach, arguing that in most cases, members of the community should be able to conduct themselves in the way that they each saw fit. These two donors rejected the implementation of legislative or clinical regulation on the assumption that it would be paternalistic in nature and would therefore interfere with their autonomy and the “independent sphere of an individual—a sphere in which they decide and act independently, and which must be protected from external interference” (57).

However, OSD is very much a new frontier in family planning in an increasingly technological age and the absence of a regulatory framework—whether community-driven or external—means that members of this community must follow the lead of earlier pioneers in this new cultural space. The lack of formal structures or rules and regulations may impact the ability of all members to participate in the community equally or to expect certain standards of behaviour. In turn, this may create space for the exploitation or abuse of newer or more vulnerable members of the community.

The donors themselves acknowledged the potential for abuse within OSD and issues around accountability. As Ed explained, many donors are aware that they have something highly sought after by recipients and some may use this as leverage “to get what they want”. JC estimated that 70%–80% of donors were sexually motivated. Whilst all the donors accepted that this was something to be expected within OSD, “a norm”, they varied in their moral interpretations of this type of behaviour. Ed felt that coercing or persuading recipients to have sex was morally inexcusable and an unnecessary part of donation, while Sam and JC felt that this should be viewed as a private matter between individuals.

JC's account stands out because the moral justification he provides for donors who attempt to elicit sexual contact from recipients draws upon the particularly pervasive “boys will be boys” cultural narrative, that excuses men for improper or poor behaviour. To some extent, in accepting that sexual motivations are to be expected within OSD, the other participants also draw upon these narratives. Weiss explains that the purpose of such narratives is “to neutralize offender culpability by blaming the questionable behaviour on mitigating circumstances, or redefining the actions as normal, not-so-bad, or justifiable due to the victims’ provocative behaviour” (58). “Boys will be boys”, in particular, suggests that stereotypical male behaviour [“courage, strong will, ambition, independence, assertiveness, initiative, rationality and emotional control”; (59)] is a natural and inevitable consequence of their biology and, as such, when a man exhibits such behaviours, even negatively, they should be neither blamed nor held to account. Women on the other hand are expected to be either sexually submissive or to avoid sexual provocation (60)—and attempts to avoid such provocation are, indeed, represented in the recipients’ stories. In effect, they should either be willing to receive sexual requests or be willing to accept that these requests are the consequence of their own behaviour. JC's views on recipient complaints are demonstrative of this. Such narratives represent a “socially approved, culturally shared language, interwoven into the belief systems of the people who invoke or honour them” (58). In the context of online sperm donation, where donors control the supply of sperm and have relatively more power than recipients to decide who to conceive with, narratives that suggest that sexually aggressive behaviour is permissible have the potential to be more potent than elsewhere.

In spite of this, the donors did not feel that OSD donors should be able to act entirely without accountability. For example, the donors said that it was fairly normal for other donors to favour anonymity in their arrangements with recipients [a finding supported by (11), and which (10), research suggests may apply particularly to donors in “opposite-sex” relationships]. The donor participants sometimes saw this as an attempt to evade responsibility for donor children or, in worst case scenarios, responsibility for poor treatment of others in the community. None of the donors in the study took an anonymous approach to donating and Ed, in particular, felt that anonymity was morally questionable in both online and clinical settings, given the right of donor children “as far as possible, to know […] their parents” (61). JC and Sam also thought that donors should take personal responsibility for their sperm donation practices and be held accountable if poor outcomes occurred if they donated indiscriminately. The extent to which donors might be willing to take responsibility for passing on STIs and genetic illnesses or care for donor children in need is uncertain, however.

### Implications of the findings

The number of people seeking to conceive via donor insemination is likely to increase to accommodate recent cultural shifts, including an increasing tendency towards delayed childbearing (62) and a growing social acceptance of “alternative” families, including same-sex/-gender parent couples and single mothers (63). This growing demand is already putting pressure on existing regulated fertility services (64) and may cause further delays in NHS waiting times. Combined with the expense of private treatments and the limited choice of identity-release donation arrangements, this is likely to result in a rise in the numbers of people turning to connection websites and social media sperm donation groups. An increasing demand for sperm may result in the strengthening of online sperm donors' positions of power within the community. As such, approaches need to be taken to (1) improve accessibility to regulated services for those who would like to take this route, and, as suggested by Gilman and Nordqvist (5), to offer a wider range of donation arrangements supported or facilitated by fertility clinics, and (2) to improve safeguarding frameworks within online sperm donation settings. It is not clear at this time what form these safeguards should take, but it is important that the owners of connection websites and social media groups, as well as recipients and donors who use OSD sites, work together on this in conjunction with other key organisations (for example, the HFEA and the Donor Conception Network).

### Strengths and limitations of the research

Whilst this exploratory research has provided new insights and further in-depth understandings of OSD donor and recipient experiences and the challenges they may face, the sample size was small, and this impacts the possibility of making generalisations. This is particularly true of the donor sample; as particularly prolific donors, their views and experiences may not be representative of those who are new to OSD or who have donated on a smaller scale. However, as more experienced donors, they were well placed to comment more generally on the norms and practices of their specific OSD communities. In addition, all the participants in this study stated they were white and all but one identified as heterosexual; further research is required to understand the experiences of groups not represented in this sample. Finally, the focus of this research was on “morally challenging” behaviour, given how little is currently known about this within OSD; however, it is important to recognise that positive experiences of OSD do also occur. An important next step is to learn more about the prevalence of problematic behaviours within these communities.

## Conclusion

The findings of this paper demonstrate that, although OSD holds many benefits for sperm donors and recipients, it also poses a number of moral challenges. Of key concern to recipients is their safety and finding a donor that they can trust, while the donors have sought to find ways to maintain autonomy in their donating practices. The participants in this study discussed experiences or observations of harm, the way in which they managed perceived risks, and the extent to which they accepted or rejected the “norms” of OSD communities. When considering the possible harms posed by OSD, it is tempting to make the case for the use of clinical services instead. However, all the participants in this study felt that OSD had an important role to play in giving people the chance to have a family and existing research has highlighted the strengths of this route to conception (5, 8). Further research is necessary to find ways to make this environment as safe and supportive for people as possible.

## Data Availability

The raw data supporting the conclusions of this article is not readily available due to participant confidentiality. Requests to access summaries of the participant narratives and further queries can be directed to the corresponding author.

## Appendix 1 Interview Schedule for Donors

*[Adapted from: “Real Life Moral Choice and Conflict Interview”*—(27)*, p. 146–147]*

Talk through the purpose and plan for the interview, run through the Participant Information Sheet [Discuss confidentiality] and ask participant to sign Consent Form. Request that the participant does not provide any identifying information for other people whilst discussing their experiences or observations.

### Part One

Many thanks for taking part in this discussion.

1. How have you found things so far? Is there anything that I can do to make you more comfortable? Do you have any questions about any aspect of the process so far?
2. What interested you in taking part in the research?
3. Could you tell me your story, perhaps from the point that you decided you wanted to donate sperm up to the present day? [apply Wengraf's *Biographical Narrative Interpretive Method* here].

### Part Two

Everyone has had the experience of being in a situation where they have had to make a decision but weren't sure of what they should do. Thinking about your experiences of interacting with sperm recipients and other online donors, can you describe an interaction or behaviour that you observed or experienced that you felt was challenging or resulted in you having to make a difficult decision?

1. What was the situation? (Be sure you get a full elaboration of the story) [apply Wengraf's *Biographical Narrative Interpretive Method* here].
2. Was there any conflict for you in that situation, such as some form of a dilemma or emotional uncertainty? Why was it a conflict?
3. In thinking about what to do, what did you consider? Why? Was there anything else you considered?
4. What did you decide to do? What happened?
5. Did you feel comfortable with this decision? Why/Why not?
6. What was at stake for you in this dilemma? What was at stake for others? In general, what was at stake?
7. How did you feel about it? How did you feel about it for the other(s) involved?
8. Is there another way to see the problem (other than the way you described it)?
9. When you think back over the experience you described, do you think you learned something from it?
10. Do you consider the situation you described as a moral problem? Why/Why not?
11. What does morality mean to you? What makes something a moral problem for you?

Repeat Part Two (1–10) if time allows.

### Part 3

1. Would you like to reflect further on anything you have said or provide any further clarification?
2. Is there anything that we have discussed that you feel you would like further advice or support on? [Signpost to relevant organisations]
3. Review positive take-home from the discussion, i.e., benefits of participating in the research or if there was an overall happy outcome from the participant's sperm donation journey.

Ask participant to complete a *Demographic and Background Form* and choose a pseudonym (if they wish to). Run through the *Participant Debrief* and write pseudonym at the top.

Thank you for taking part in this discussion.

**Note to Interviewers: Questions should follow references to judgments about the situation. Follow any references to feelings that are mentioned, e.g., Why did you feel mad or angry? Also follow moral language, i.e., should, ought. Questions should focus on: In whose terms are judgments made? Try to understand the terms of the self and the self's perspective on the terms of the other.

## Appendix 2 Interview Schedule for Recipients

*[Adapted from: “Real Life Moral Choice and Conflict Interview”*—(27)*, p. 146–147]*

Talk through the purpose and plan for the interview, run through the Participant Information Sheet [Discuss confidentiality] and ask participant to sign Consent Form. Request that the participant does not provide any identifying information for other people whilst discussing their experiences or observations.

### Part One

Many thanks for taking part in this discussion.

1. How have you found things so far? Is there anything that I can do to make you more comfortable? Do you have any questions about any aspect of the process so far?
2. What interested you in taking part in the research?
3. Could you tell me your story, perhaps from the point that you decided you wanted to have a baby up to the present day? [apply Wengraf's *Biographical Narrative Interpretive Method* here]

### Part Two

Everyone has had the experience of being in a situation where they had to make a decision but weren't sure of what they should do. Thinking about your experiences of interacting with sperm donors that you have met online, can you describe an experience that you felt was less than positive and resulted in you having to make a difficult decision?

1. What was the situation? (Be sure you get a full elaboration of the story) [apply Wengraf's *Biographical Narrative Interpretive Method* here].
2. Was there any conflict for you in that situation, such as some form of a dilemma or emotional uncertainty? Why was it a conflict?
3. In thinking about what to do, what did you consider? Why? Was there anything else you considered?
4. What did you decide to do? What happened?
5. Did you feel comfortable with this decision? Why/Why not?
6. What was at stake for you in this dilemma? What was at stake for others? In general, what was at stake?
7. How did you feel about it? How did you feel about it for the other(s) involved?
8. Is there another way to see the problem (other than the way you described it)?
9. When you think back over the experience you described, do you think you learned something from it?
10. Do you consider the situation you described as a moral problem? Why/Why not?
11. What does morality mean to you? What makes something a moral problem for you?

Repeat Part Two (1–11) if time allows.

### Part 3

1. Would you like to reflect further on anything you have said or provide any further clarification?
2. Is there anything that we have discussed that you feel you would like further advice or support on? [Signpost to relevant organisations]
3. Review positive take-home from the discussion, i.e., benefits of participating in the research or if there was an overall happy outcome from the participant's sperm donation journey.

Ask participant to complete a *Demographic and Background Form* and choose a pseudonym (if they wish to). Run through the *Participant Debrief* and write pseudonym at the top.

Thank you for taking part in this discussion.

**Note to Interviewers: Questions should follow references to judgments about the situation. Follow any references to feelings that are mentioned, e.g., Why did you feel mad or angry? Also follow moral language, i.e., should, ought. Questions should focus on: In whose terms are judgments made? Try to understand the terms of the self and the self's perspective on the terms of the other.
